# Turning date palm fronds into biocompatible mesoporous fluorescent carbon dots

**DOI:** 10.1038/s41598-018-34349-z

**Published:** 2018-11-02

**Authors:** T. Kavitha, S. Kumar

**Affiliations:** Department of Mechanical and Materials Engineering, Khalifa University of Science and Technology, Masdar Institute, Masdar City, P.O. Box 54224, Abu Dhabi, UAE

## Abstract

Here, we demonstrate the synthesis of mesoporous carbon dots (Cdot) from date palm fronds and their excellent excitation wavelength-independent photoluminescence (PL), with high photo- and storage-stability, superior biocompatibility and thermal and electrical conductivity for the first-time by a simple, green, one-step carbonization method. Interestingly, the as-obtained Cdot manifest the spherical shape of about 50 nm average diameter having surface mesopores of size less than 10 nm with *sp*^2^ hybridized carbon. The as-synthesised mesoporous Cdot, first of its kind, evince yellow-green PL (preferred over blue PL for biological applications) around 450 nm under excitation wavelength range of 320–420 nm with absolute quantum yield of 33.7% exhibiting high photo- and storage-stability. The thermal and electrical conductivity of Cdot/water nanofluids without any surfactants is illustrated. Application of Cdot as interfacial material in organic photovoltaic cell is manifested. The Cdot exhib visible sunlight driven photocatalytic and antibacterial activity. Mesoporous Cdot further reveal excellent biocompatibility with fibroblast cell (greater than 95% viability). The novelty of this study in the formation of multifunctional mesoporous Cdot from date palm fronds could inspire both research and industrial interests in the synthesis of biomass-derived Cdot and their application in a wide array of fields.

## Introduction

As a new member of carbon family and potential alternative to classical metal based lethal semiconductor quantum dots and organic dyes^1^, carbon dot has extraordinary benefits like simple and convenient synthesis and functionalization, prominent biocompatibility, colourful and stable photoluminescence, low cost and good resistance to photo and chemical degradation^2–6^. Carbon dots (Cdot) serve as benign candidates as fluorescent probes in bioimaging, photocatalysts, bio- and chemical -sensors and in photovoltaics and optoelectronics^7–13^. A series of methods including laser irradiation, electrochemical itching, microwave/ultrasonic preparation, hydrothermal/acidic oxidation routes, arc discharge and plasma treatment^14–19^, have been used to synthesize Cdot. However, many of these preparation methods use expensive or toxic starting materials, high temperature, long reaction time and surface-passivation. They are also environmentally unsafe due to the use of alkali or acid to remove excess oxidizing agent^20–22^. Hence a simple, green and energy -efficient way to synthesize Cdot is highly desirable.

In the above context, applying green chemistry principles to synthesize Cdot from biomass would be more efficient in terms of biomass utilization and waste minimization as it involves one or few steps and do not call for separation of the intermediates^23^. The biomasses studied till date are orange juice, banana juice, soybeans, grass, corn stalk, glucose, chitosan, chitin, agricultural biomass, pigeon feathers, egg and manure^24–29^. The biomass of our interest is date palm fronds (leaves and rachis) as it has quite higher lignin content of 25 g/100 g of biomass as biomass with higher lignin content is more favourable for the generation of high quality carbon material. Thus use of date palm fronds biomass as carbon source to synthesize Cdot could be promising as it is a renewable feedstock with natural, nontoxic and biocompatible properties.

The most studied property of Cdot is their inherent photoluminescence and most of Cdot show intense emission only at blue light spectrum while they show very weak emission at the longer wavelength (yellow to red light spectra). This hampers its real time application as fluorescent probes in bioimaging and other biological applications given that blue light emission would be significantly interfered by the common blue auto fluorescence of the biological matrix and potential photo damage by ultraviolet excitation light to biological tissues^9,30^. Hence for real time utilization of Cdot in bio-applications, preparation of Cdot that illustrate strong emission other than blue light spectrum is highly desirable.

The efficient capture of solar energy is the need of the hour in meeting the energy needs of the future. With respect to this, Cdot are finding application in photocatalytic solar energy conversion due to their intrinsic properties like good solar spectrum utilization, fast migration of charge carriers, efficient surface redox reactions and excellent performance with long term stability during operation without poisoning^31^. The photo induced redox processes have made Cdot an excellent candidate as photo-activated antibacterial agents to bacterial cells, and photocatalyst particularly useful under visible/natural light illumination^32^. Mesoporous materials have exceptional properties like ultra-high surface areas, large pore volumes, tunable pore sizes and shapes and also exhibit nanoscale effects in their mesochannels and on their pores as well as on their pore walls. These features are particularly advantageous for applications in energy conversion and storage like water splitting devices, solar cells and batteries^33–35^. Eventhough there is an extensive study on photoluminescent, biological, photovoltaic, optoelectronic and photocatalytic property of Cdot, till date there is no study on thermal and electrical properties of the Cdot. The enrichment of thermal conductivity of conventional fluids used in heat exchanger (water, ethylene glycol, silicone oil) by the suspension of solid particles is not achievable practically due to disadvantages like sedimentation, erosion, fouling and increased pressure drop of the flow channel^36^. Nanofluids can be considered as next generation heat transfer fluids as they offer exciting new possibilities to enhance heat transfer performance compared to pure liquids. We report for the first time, study on thermal and electrical conductivity of Cdot based nanofluids with water as base fluid without any surfactant.

Herein, we report for the first time synthesis of Cdot by a simple, chemical free (without any additive like salts, acids or bases), one step carbonization method for biomass date palm fronds. The as -prepared Cdot manifest spherical shape of size ~50 nm with mesoporous attributes. The mesoporous Cdot show green yellow fluorescence with higher photo and storage stability. The thermal and electrical conductivity of mesoporous Cdot nanofluids in water without any surfactant at different temperature and concentration have been studied for the first time and the results have been discussed. We demonstrated its application in organic photovoltaic cells. Mesoporous Cdot are bestowed with visible light driven photocatalytic and antibacterial activity. The as -prepared mesoporous Cdot show excellent biocompatibility against fibroblast cell.

## Experimental

### Materials

The date palm fronds consisting of both leaves and woody stem were collected from Masdar City landscapes in Abu Dhabi, UAE. Collected samples were air dried at room temperature resulting in dry matter (total solids) content of approximately 92%. Dried samples were crushed to particle size of less than 2 mm using IKA_ Werke MF 10.1 mill. Crushed samples were stored in air tight plastic bags until use. High glucose Dulbecco’s modified Eagle’s medium (DMEM), fetal bovine serum (FBS), phosphate buffer saline (PBS), (3-(4,5-dmethylthiazol-2-yl)−2,5-diphenyltetrazolium bromide (MTT, 98%), were bought from Sigma Aldrich and used without any purification. Deionized water was used through the whole study.

### Synthesis of carbon dots (Cdot)

Cdot were synthesized by carbonization of date palm fronds at 300 °C for couple of hours at a heating rate of 10 °C/min in a limited supply of air. After carbonization, the biochar was mechanically ground to fine powder, dispersed in water, centrifuged and the supernatant containing Cdot were collected and used for further studies.

### Characterization

The morphological characterization of the date palm fronds and Cdot were conducted using a Quanta 250 ESEM, UK. The samples were coated with Au by ion sputtering at 10 nm thickness to provide the conducting path for optimum examination of the surfaces. A high resolution transmission microscopy (HRTEM) study of Cdot was also carried out using a Tecnai TF20, 200 kV instrument. Dynamic light scattering (DLS) and zeta potential measurements were performed using a Zeta-Meter System 4.0. The UV-Vis and photoluminescence (PL) spectrum were recorded on a UV/Vis spectrophotometer thermo scientific genesys 10 S and LS-55 fluorescence spectrometer, respectively. The photobleaching measurement was performed with a LS-55 fluorescence spectrometer using the built-in light source. The quantum yield of the as-synthesized Cdot is calculated using quinine sulphate as standard using the following equation:

$$Q{Y}_{s}=Q{Y}_{st}(\frac{{A}_{st}}{{I}_{st}})(\frac{{I}_{s}}{{A}_{s}}){(\frac{{\eta }_{s}}{{\eta }_{st}})}^{2}$$

where $QY$ is quantum yield, $I$ is the integrated emission intensity, $\eta$ is the refractive index, and $A$ is the optical density, where $s$ refers to Cdot and $st$ to standard reference quinine sulphate^30^. The thermal diffusivity of Cdot/water suspension was determined via laser-flash thermal diffusivity technique using Flash Diffusivity TA DXF-EM900 operated with Xenon lamp. All the measurements were taken after calibrating the instrument with water. The electrical resistance characterization of Cdot/water suspension was measured with Tektronix DMM 4050 multimeter. Cdot were probed for their semi conductive behaviour using probe station (CASCADE, USA). The sample was drop casted onto glass substrate and two-contact method was used for the measurement. The current was measured as a function of voltage using two probe method.

### Photocatalytic Degradation of MO Dye

Methyl orange (MO) dye was used as a probe molecule to evaluate the photocatalytic activity of Cdot. The photocatalytic reaction was conducted under sunlight. In a typical experiment, 0.1 mg of MO dye was added into the water (10 ml) containing 0.1 mg of Cdot, followed by the addition of 1 ml H_2_O_2_. Prior to the irradiation, the suspension was magnetically stirred in the dark for 30 min to establish the adsorption/desorption equilibrium of MO. A 2 ml of the sample was withdrawn for every 1 hr. Before analysis, the suspension was centrifuged to remove any suspended solid catalyst. The residual concentration of dye was measured using a Jasco V-650 UV–visible spectrophotometer.

### Antibacterial Studies

Bactericidal activities of Cdot were evaluated against *E. coli*. In 96-well plates in two different set of experiments. In first experiment, the well plate was added with bacterial suspension (cell density of 10^6^ CFU/ml) of around 170 μL and 30 μL Cdot at different concentrations (0 to 100 μg/ml). The plates were exposed to room light from 50 W LED light mounted on the 8-feet ceiling in the laboratory. Viable cell numbers of both control and treated plated were determined via optical density measurement method after 16 hrs. In second experiment, the cells were treated with an optimized (from first experiment) concentration of Cdot for various time intervals to analyze their growth procedure in comparison to control. Rest of the procedure was same.

### Biocompatibility Studies

The NIH-3T3 (mouse embryonic) fibroblast cell line obtained from American Type Culture Collection (ATCC) was cultured in DMEM supplemented with 10% FBS at 37 °C under 5% CO2 and 95% relative humidity. The cells (1 × 10^5^) were seeded into a 4-well plate and incubated for 24 h. Then, 100 μL of a fresh aqueous solution containing Cdot (10 or 100 mg/L) was added into cells containing plate with 1 mL culture medium. After 1, 3, or 5 days, MTT (100 μL) was added to each well and incubated at 37 °C for 4 h. All media were removed and then 150 µL of DMSO was added and kept for 15 min in the dark. The absorbance was measured at 570 nm using a Bio-RAD model 1680, Microplate reader with pure DMSO as a blank. A non-treated cell was used as a control and the percent cell viability was calculated using A_test_/A_control_ × 100, where A_test_ and A_control_ are the absorbance’s of the wells (with the Cdot) and control (without the Cdot), respectively.

## Results and Discussion

**Figure 1:**
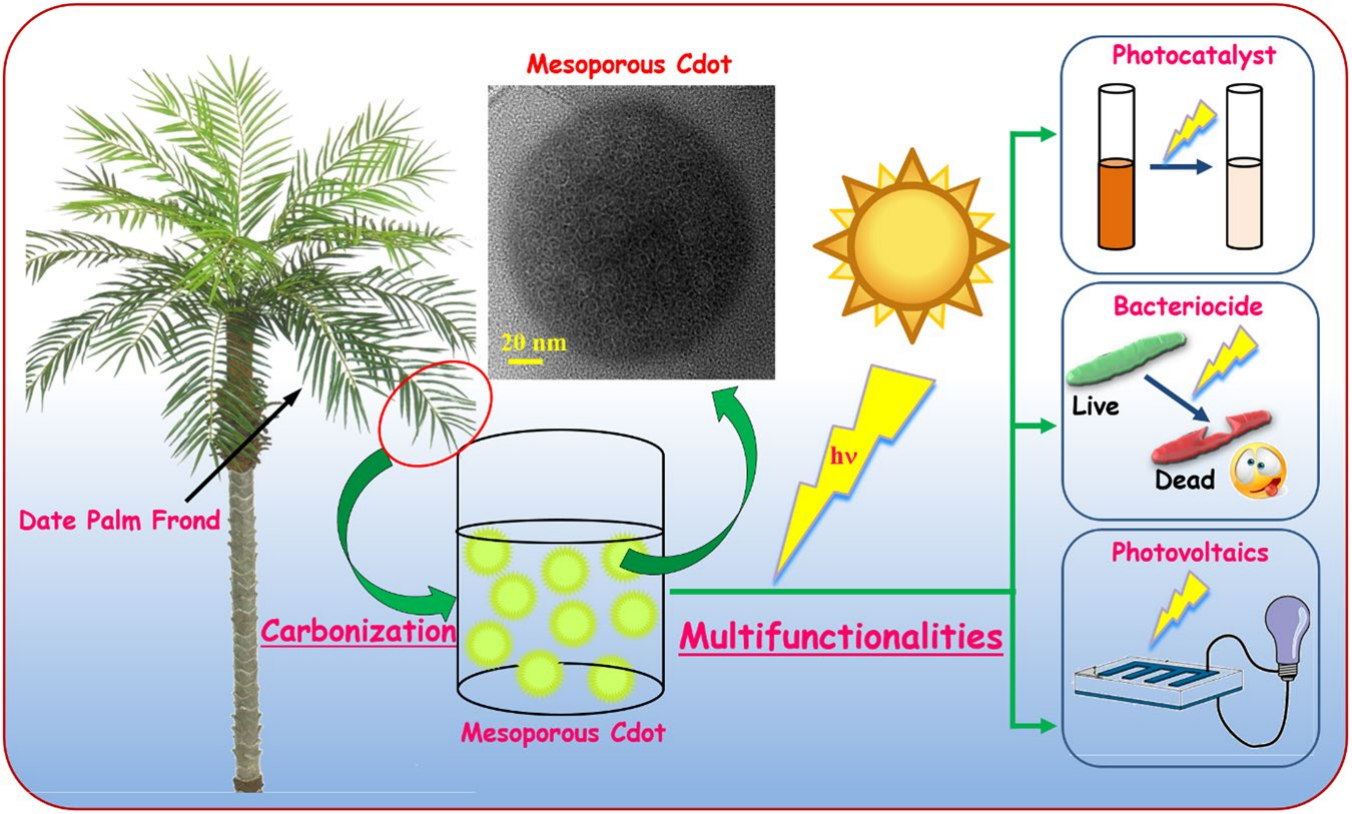
Schematic depicting the synthesis of Cdot from date palm fronds.

We present a very simple, one-step carbonization route to synthesize Cdot from date palm fronds for the first time as depicted in Fig. 1. During carbonization, the carbonaceous material in date palm fronds gets oxygenated which is an important prerequisite for Cdot synthesis. In general, aliphatic carbon moieties are more reactive than aromatic carbon, so the hemicellulose and cellulose more easily decompose leading to defects^37^. But in our case due to higher aromatic lignin content we were able to achieve more stable and defect-free Cdot. In this method, no strong acid solvent, or chemical reagent or surface passivation reagent was used and it was purely based on green chemistry principles. The merits of one -pot synthesis of the Cdot are:

i. Date palm fronds, a sustainable source of precursor for Cdot synthesis.
ii. Being a natural material., it acts as a biocompatible source.
iii. Absence of toxic chemicals/ reactants.
iv. Ideal for commercial synthesis of Cdot as the method is fast.
v. Biomass utilization and waste minimization.

**Figure 2:**
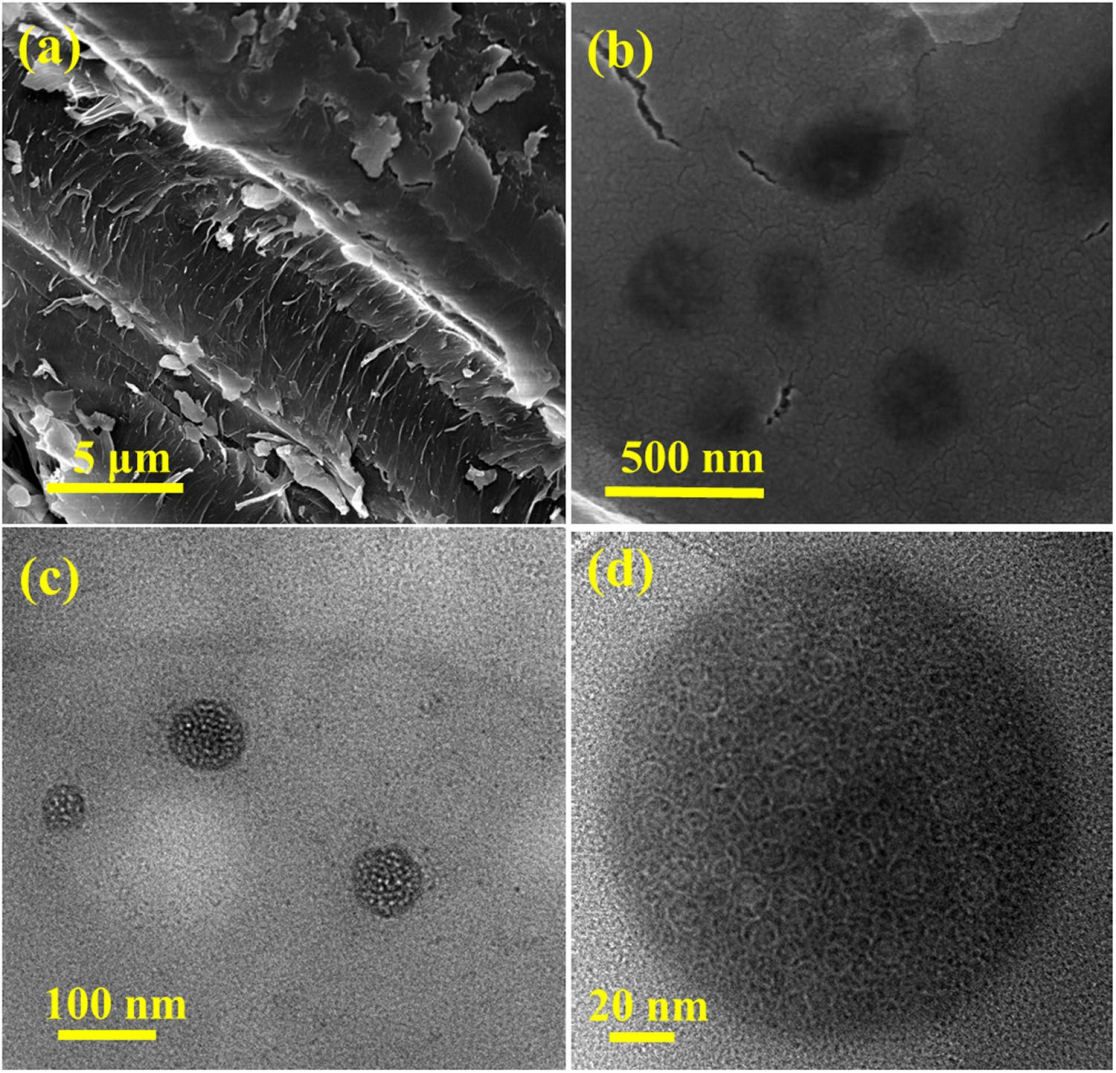
(**a**) SEM image of biomass date palm fronds, (**b**) SEM, (**c**) TEM and, (**d**) HR-TEM images of as-synthesized Cdot.

The morphology of date palm fronds is seen under scanning electron microscopy (Fig. 2a) and it shows a rigid surface of lignocellulose structure of wood with cellulose base embedded within the matrix of cross-linked hemicellulose and lignin^38^. After carbonization, date palm fronds are converted into Cdot with spherical morphology (Fig. 2b). The structure of Cdot is ascertained in more detail by transmission electron microscopy as shown in Fig. 2c. Cdot are uniform in size, well dispersed having an average diameter of 35 nm. A dynamic light scattering study also confirms the monodisperse nature of Cdot with hydrodynamic diameter of 1650 nm. High resolution TEM image (Fig. 2d) exposes the mesoporous nature of Cdot and the absence of lattice fringes exemplifies their polymer like amorphous nature^39^. The mesopores on the surface of Cdot is first of its kind and the mesoporous Cdot reveal a tremendous potential and impact on a large number of functional applications, such as catalysis, energy storage, nanomedicine, optics, sensing and adsorption.

**Figure 3:**
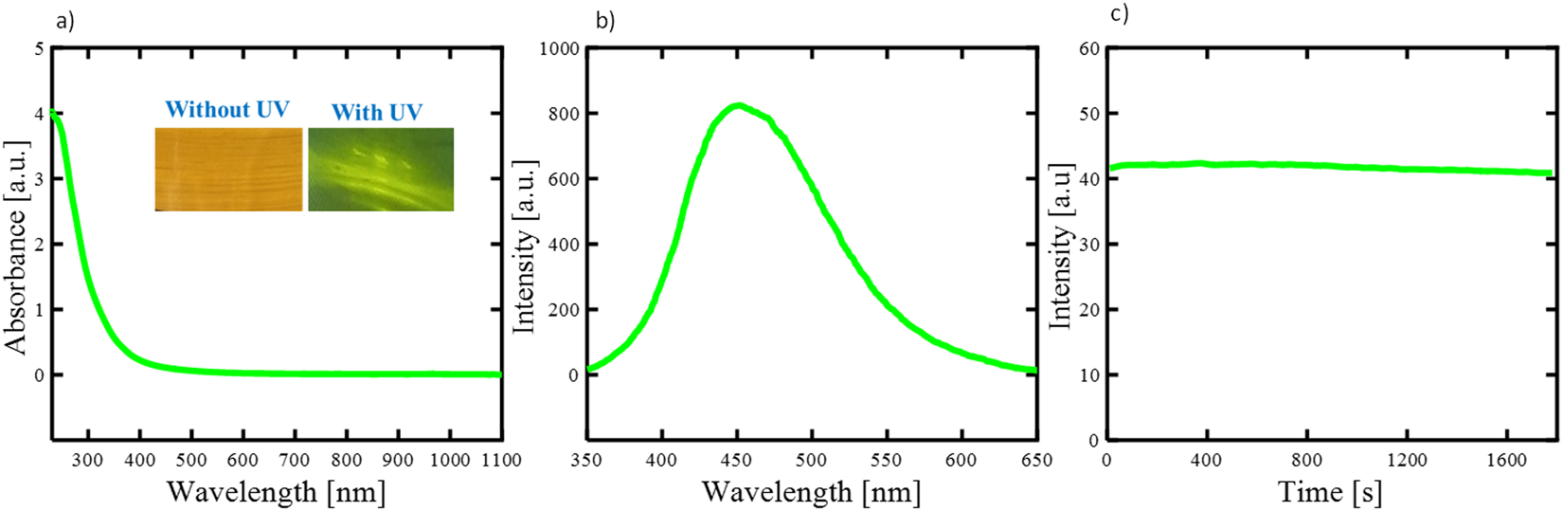
(**a**) UV-vis spectrum of Cdot (inset-photograph of Cdot aqueous solution excited by visible light and UV lamp), (**b**) photoluminescence emission spectra of Cdot at excitation wavelength of 245 nm and (**c**) photo decay spectrum of Cdot illuminated for 30 min.

Figure 3 illustrates the optical properties of the Cdot. The UV-vis absorption spectrum of Cdot in water manifests a vivid absorption peak located at 245 nm, which could be typically assigned to the π-π* transition of the aromatic *sp*^2^ domains from the carbon core of fluorescent Cdot^40,41^. There are no other carbonaceous materials, which usually get absorbed at higher wavelengths^42^ and hence it is clear that Cdot obtained from date palm fronds are of high purity. The surface of Cdot is literally neutral as demonstrated by zeta potential which confirms the absence of functional groups or defects on the surface. These findings explain that our mesoporous Cdot consist of π-conjugated domains in their carbon cores and amorphous regions on their surfaces. The band gap between the energy states in our Cdot is calculated to be 5.01 eV which explains their semiconducting nature. The inset in Fig. 3a demonstrates that the as -prepared Cdot in aqueous solution exhibiting brown colour under visible light while it spectacled greenish yellow fluorescence when excited under UV light. When excited at the maximum excitation wavelength of 245 nm, the mesoporous Cdot evince a strong emission peak centred at 450 nm which could be based on the band gap transitions in the conjugated π-domains^43^. Our Cdot exhibit excitation wavelength- independent emission feature unlike most of the previously reported Cdot i.e. it shows only a greenish yellow fluorescence regardless of the excitation wavelength which bestows only one type of predominant emission state present in the mesoporous Cdot. The fluorescence decay of Cdot monitored at 450 nm with continuous illumination for 30 mins reveals the high photostability of our Cdot as there is no noticeable loss in the emission intensity. The quantum yield of the mesoporous Cdot in water is 33.7% under the excitation of 245 nm.The yellow green fluorescence of our Cdot with mesoporous surface could open new avenue for above mentioned functional applications with safe in situ monitoring for cells in bio related applications.

**Figure 4:**
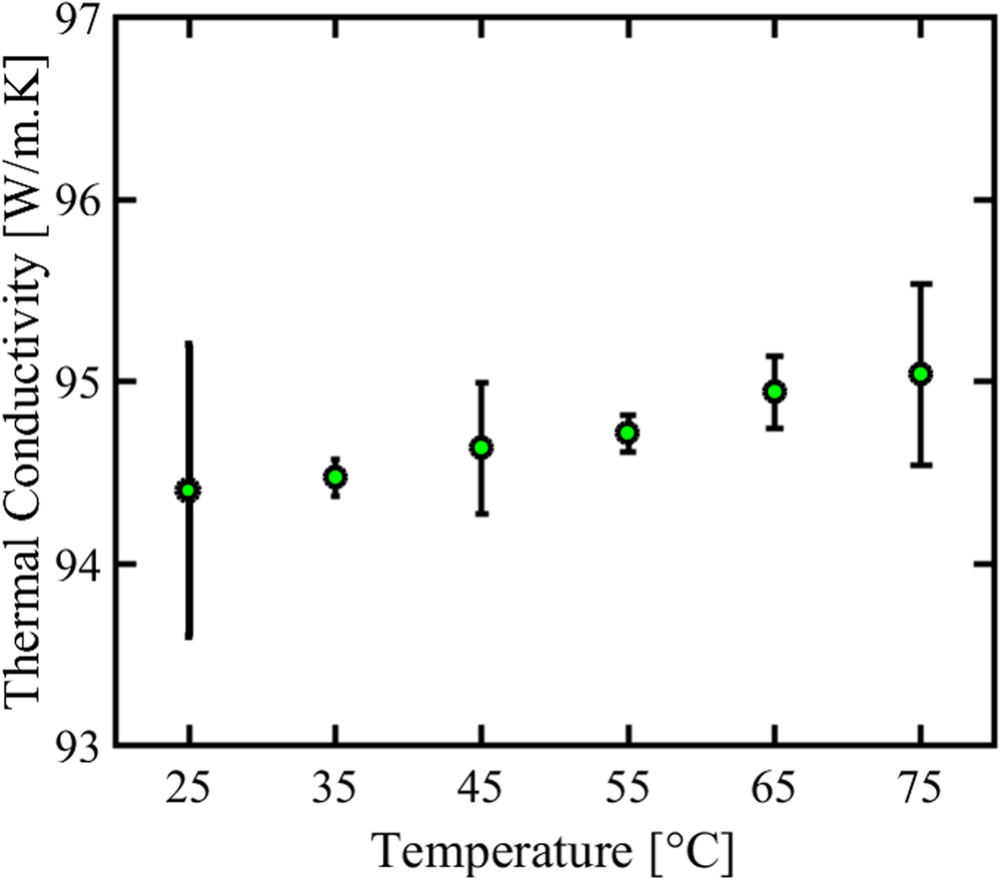
Thermal conductivity measurement of water based Cdot nanofluids at different temperatures.

**Figure 5:**
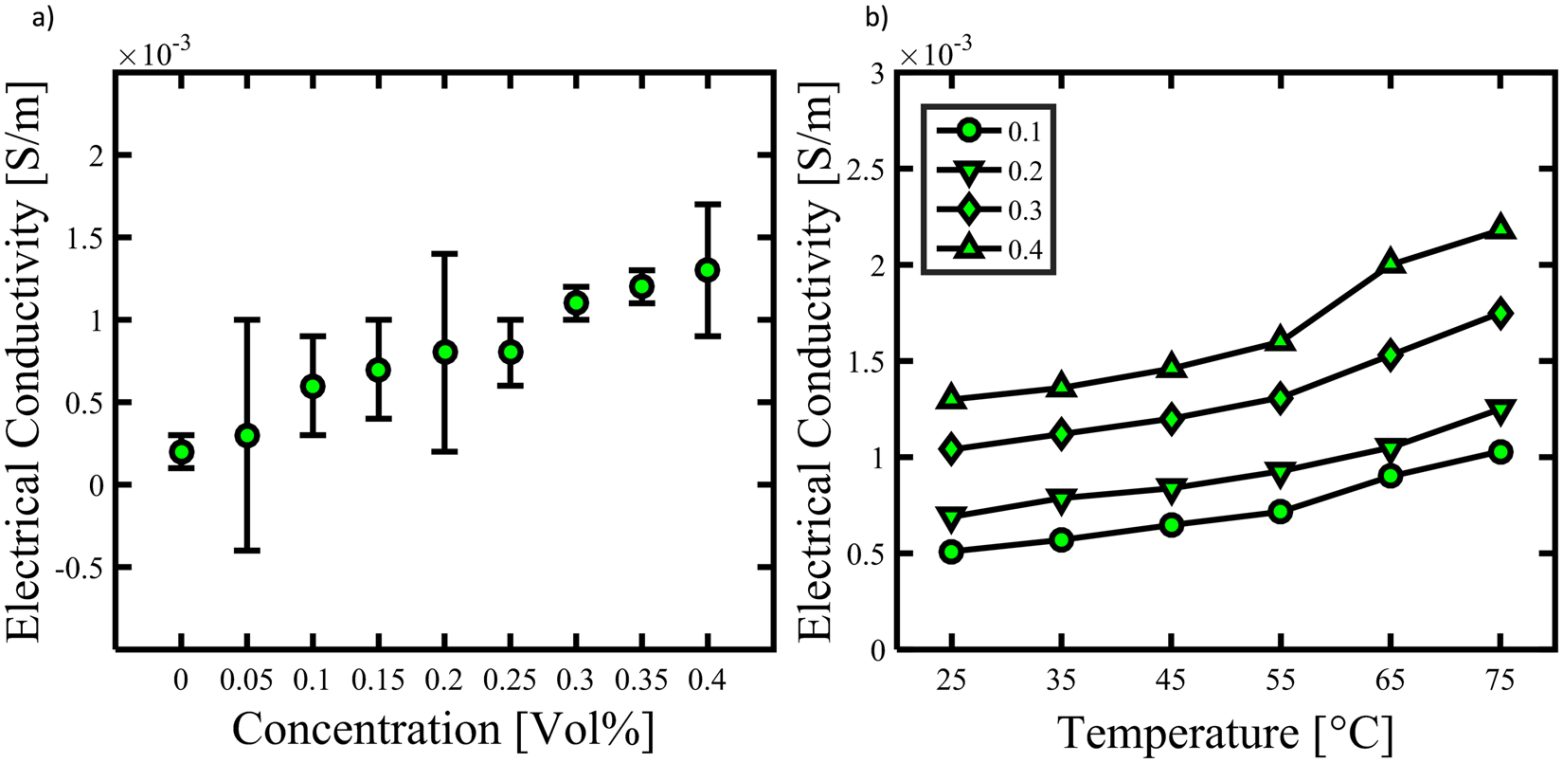
Electrical conductivity of water based Cdot nanofluids (**a**) for different volume fraction at room temperature and, (**b**) for different volume fraction at varying temperature.

The thermal conductivity of mesoporous Cdot dispersed water samples (0.4 vol.%) was measured at different temperatures ranging from 25 to 75 °C as illustrated in Fig. 4. The thermal conductivity increases in an approximately linear fashion with increasing temperature: at 25 °C it is 94.4 W/m.K whereas at 75 °C it increased to 95.07 W/m.K. The thermal conductivity of water at 25 °C is estimated to be 0.6 W/m.K which clearly demonstrates the role of Cdot in thermal conductivity of water based Cdot nanofluids. Eventhough thermal conductivity of Cdot is not high as compared to other members of carbon family like CNTs and graphene, the thermal conductivity of Cdot is first ever studied and reported. Apart from phonon transfer, the mechanism for thermal conductivity of our mesoporous Cdot/water could be Brownian motion of suspended Cdot in water which eventually increases the energy transport inside the nanofluids and thus thermal conductivity^44^. In addition, good and stable dispersion of Cdot in water along with surface and quantum effects of mesoporous Cdot could also be a prime reason for thermal conductivity of nanofluids^45,46^. We also studied the electrical conductivity of Cdot/water nanofluids having different volume fractions of Cdot and at different temperatures as shown in Fig. 5a,b. The electrical conductivity of Cdot/water nanofluids increases with the increase in volume fraction of Cdot in approximately linear fashion. The electrical conductivity of water is about 0.0002 S/m whereas the electrical conductivity of Cdot/water nanofluids increases to above 0.0013 S/m for a volume fraction of 0.4 vol.% which is ~300% more than the water. With increasing volume fraction as well as increasing temperature, the electrical conductivity increases. At 75 °C, the electrical conductivity increases from 0.001 S/m to 0.002 for S/m for 0.1 to 0.4 vol. % respectively. The conducting path ways of Cdot/water nanofluids could be due to electrophoretic mobility together with ion-cloud that attributes to electrical double layer interactions^47^. The electrokinetic interactions dictate the distributions of the electric potential, ionic densities, and fluid velocity in the nanofluid which in turn determine the current density distribution and transport properties such as the electrophoretic mobility of the Cdot and the electric conductivity of the Cdot suspended nanofluid. Moreover, Cdot are porous, that is, permeable to the fluid and ions. Therefore, the electrophoretic mobility of porous spherical Cdot under internal flow field contributes to the effective electrical conductivity of the water based Cdot nanofluid^48^. Thus, with increasing volume fraction, the overall electrical conductivity of the nanofluids increases. In addition the 3D nanometre size framework can bring in nanoscale effects which bestow mesoporous Cdot with this electrical conductivity^46^. These findings on thermal and electrical conductivity of Cdot/water nanofluids could open new windows for cost effective Cdot based nanofluids for various new potential applications other than bioimaging and sensing.

**Figure 6:**
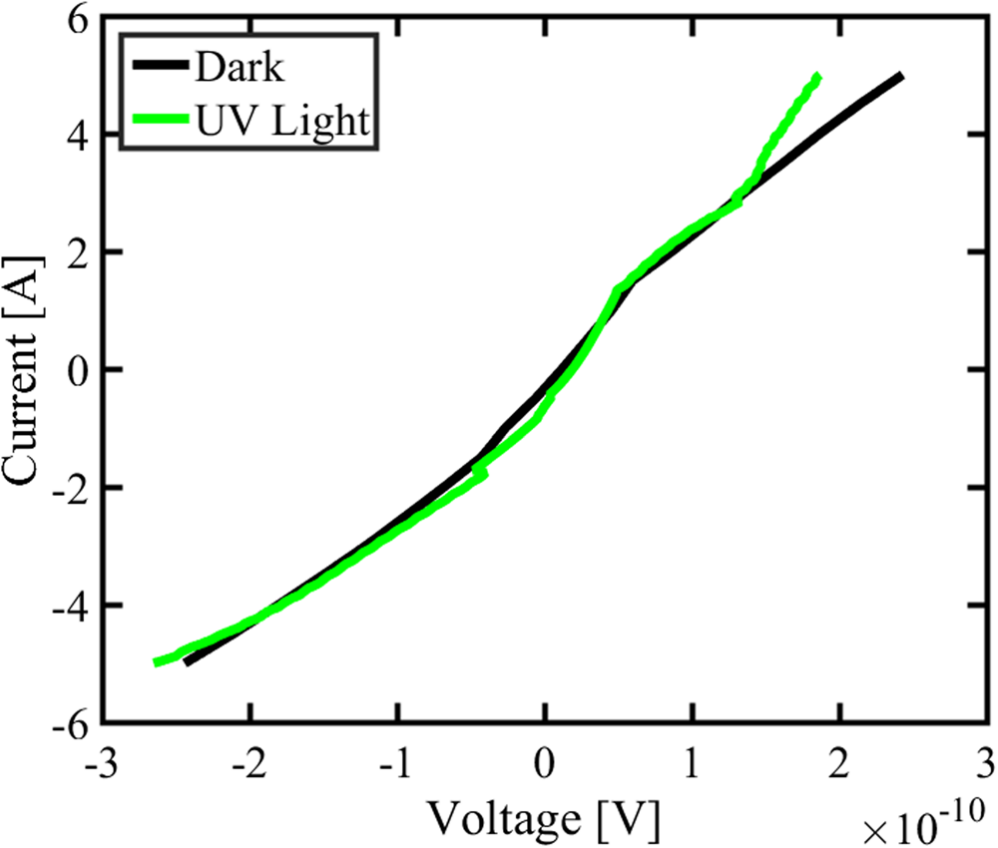
Dark and light I-V characteristics of Cdot.

As shown in Fig. 6, the dark as well as UV light I-V characteristics of the Cdot exhibit the ohmic behaviour and there is no appreciable change in the electrical characteristics during UV exposure. The Cdot electrical conductivity of ∼2 × 10^–3^ S/m is quite comparable to the electrical conductivity of interfacial transport layers used in organic and hybrid solar cells. For example, poly (3,4 ethylenedioxythiophene): poly(4-styrenesulfonate) (PEDOT:PSS) has electrical conductivity of ∼4.4 × 10^–3^ S/m. Therefore, carbon dot is promising as low-cost interfacial material option for organic and hybrid solar cells and can significantly reduce the cost of Organic photovoltaic (OPV) technology.

**Figure 7:**
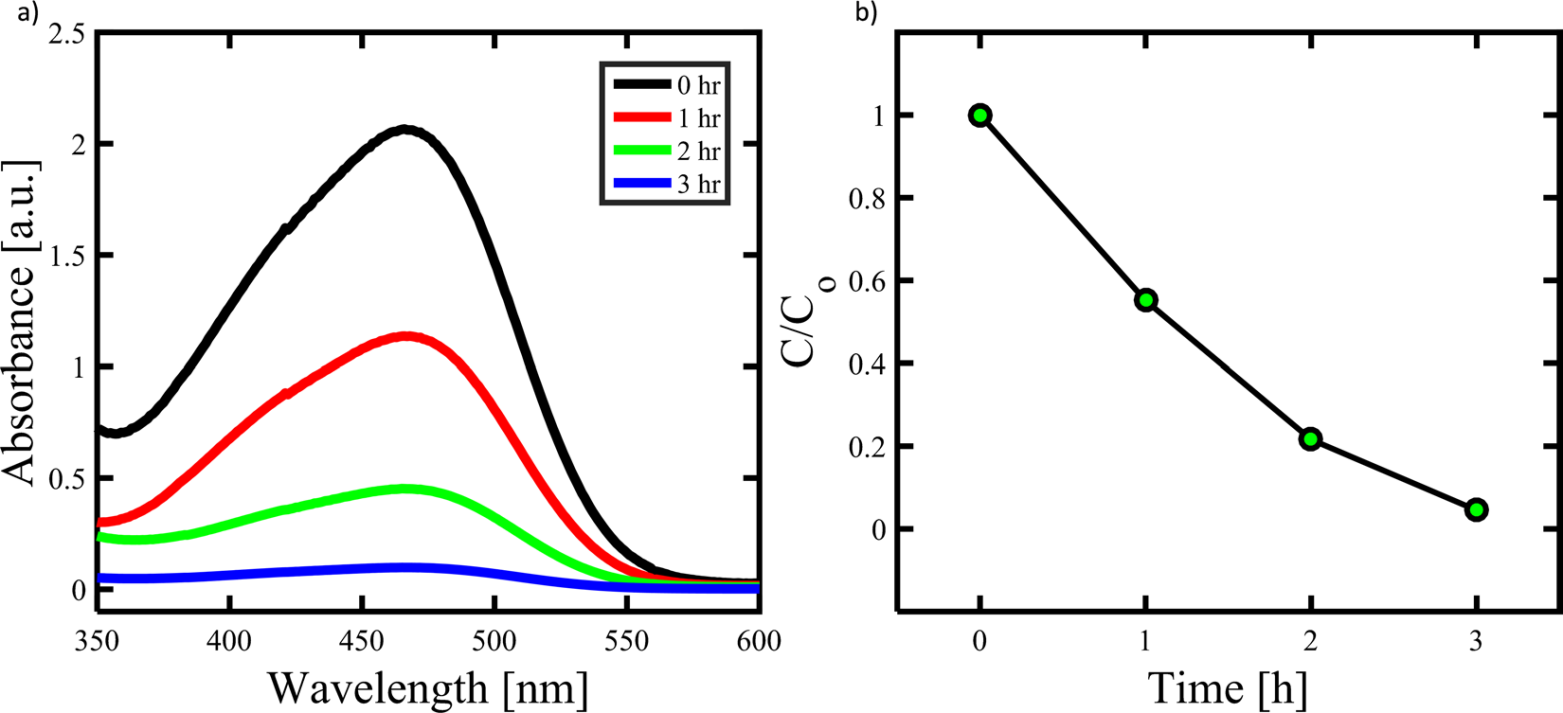
(**a**) UV–visible spectra of the MO solution in the presence of Cdot at different UV-irradiation time, and (**b**) photodegradation of the MO dye.

Photocatalysis is a green technique that has been widely applied in the fields of environmental remediation and solar water splitting^49^. Figure 7a shows the typical time-dependent UV-vis absorption spectra of the MO dye solution during the photodegradation in the presence of Cdot with the aid of H_2_O_2_. It is seen that dye exhibited a maximum absorption peak at around 470 nm. Note that the intensity of absorption spectra decreases gradually with increasing the irradiation time, indicating that a strong oxidation of dye has been occurred in the presence of Cdot under sunlight irradiation. All these observations indicated that Cdot exhibited excellent performance for the degradation of dye. Figure 7b shows the photocatalytic performance of Cdot for the degradation of MO dye in the presence of H_2_O_2_ with time. Approximately 50% of the dye was degraded by the Cdot within 1 hr. However, the Cdot exhibited no obvious photocatalytic activity in the absence of H_2_O_2_ (data not shown). The possible mechanism for the degradation of MB dye may be proposed as follows: H_2_O_2_ molecules adsorbed on Cdot would be photodecayed into active oxygen species (HO), which have strong oxidation ability to photodegrade the MO dye into CO_2_, and H_2_O through a series of redox reactions. In addition, a strong π–π interaction exists between mesoporous Cdot and MO dye which results in maximum adsorption of dye onto mesoporous structure that aids the catalytic process. Cdot acts as both electron donor and acceptor. Under sunlight irradiation, excellent light harvesting capabilities as well as unique photoinduced electron transfer facilitate the photocatalytic decomposition of MO dye^50^.

**Figure 8:**
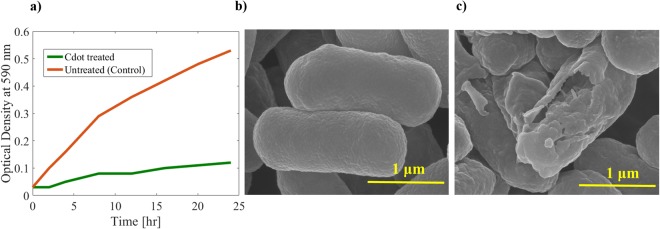
(**a**) Growth behaviour of *E. coli* after treatment with and without 70 μl/ml of Cdot for various treatment times in presence of light. (**b**,**c**) SEM images of E.Coli before and after treatment of 10 h.

Figure 8a shows the growth behaviour of *E. coli* with and without treatment of Cdot (70 µg/ml) for various time intervals. It is interesting to see that growth of bacterial cells was reduced from very start and even after 24 h, the number of *E. coli* were very small compared to control under light irradiation. Without light there was no pronounced antibacterial activity of Cdot (results not shown here), rather there was noticeable growth which could be due to biomass precursor. The SEM images (see. Fig. 8(b and c)) of E.coli treated with Cdot for 10 h under light indicate that the cell membrane was wrinkled and ruptured with severe change in morphology of cell in contrast to E.coli without treatment having smooth surface with intact morphology suggesting cell death. The obtained results confirm that Cdot reduce the growth of pathogens specifically under visible light illumination. The mechanism for apoptosis of bacteria could be explained as follows: the photo induced redox species and emissive excited states could lead to the bactericidal functions^32^, similar to quantum dots, Cdot transfer energy directly to molecular oxygen to generate singlet oxygen resulting in reactive oxygen species production and thus cell death.

**Figure 9:**
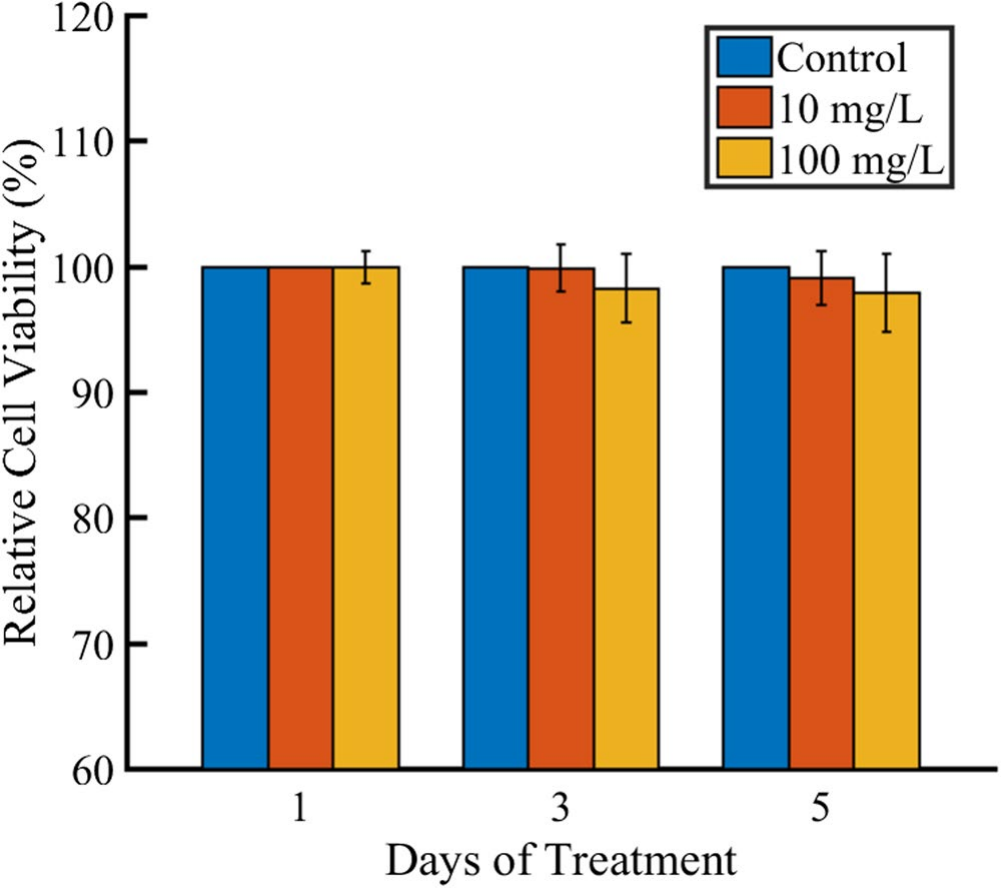
*In vitro* cytotoxicity evaluation of the mesoporus Cdot using MTT assay against fibrobalst cells.

To evaluate the biosafety of mesoporous Cdot, MTT assay were performed against fibroblast cells with 0 (control), 10 and 100 mg/L Cdot for 1, 3 and 5 days as demonstrated in Fig. 9. The cell viability did not show any statistically significant differences. After 24 h of incubation, there was literally no change in cell viability even for a concentration of 100 mg/L. Even after 5 days of incubation the cell viability was greater than 95% which signifies its excellent biocompatibility. Importantly, the concentrations used in this *in vitro* study are much higher than those used in potential applications (20 μg/ml) such as optical imaging of live cells^51^. The results confirm the low toxicity, excellent biocompatibility and safety in *in vitro* and *in vivo* applications of Cdot in bioimaging (being bestowed with highly stable greenish yellow fluorescence) and also drug delivery applications due to mesoporous structure.

## Conclusions

We report a simple, green, cheap, ingenious, rapid, convenient and eco-friendly synthetic route to convert date palm fronds with inherent cross-linked lignocellulose structure into high value added Cdot by a simple carbonization approach. Amorphous Cdot with average diameter of 50 nm with mesoporous structure on their surface were synthesized. The mesoporous Cdot derived from date palm fronds is first of its kind. The as-prepared mesoporous Cdot exhibited greenish yellow excitation wavelength-independent fluorescence, excellent photo and storage stability and extraordinary biocompatibility. The thermal and electrical conductivity of Cdot/water nanofluids without any dispersant was studied for the first time. We demonstrated its application as low cost interfacial material for organic photovoltaic cells. The Cdot also showed enhanced photocatalytic activity towards MO degradation under sunlight. Cdot exhibit light activated biocidal functions. In the near future, apart from bioimaging and bioanalytical applications, biomass derived mesoporous Cdot could emerge as a promising candidate for applications such as energy conversion and storage, optoelectronics, photocatalysts, heat exchangers, bacteriocide, information encryption and electrical chemistry. This synthesis approach based on green chemistry principles demonstrated here could inspire future research utilizing renewable biomass to produce multifunctional carbon nanomaterials in large scale.
